# Recent advances with optical upconverters made from all-organic and hybrid materials

**DOI:** 10.1080/14686996.2019.1610057

**Published:** 2019-05-28

**Authors:** Roland Hany, Marco Cremona, Karen Strassel

**Affiliations:** a Empa, Swiss Federal Laboratories for Materials Science and Technology, Laboratory for Functional Polymers, Dübendorf, Switzerland; b Optoelectronic Molecular Laboratory, Physics Department, Pontifical Catholic University of Rio de Janeiro, Rio de Janeiro, Brazil; c Institute of Chemical Sciences and Engineering, Ecole Polytechnique Fédérale de Lausanne, Lausanne, Switzerland

**Keywords:** Upconverter, photodetector, near-infrared, organic light-emitting device, NIR imaging, 40 Optical, magnetic and electronic device materials, 204 Optics / Optical applications

## Abstract

The growing interest in near-infrared (NIR) imaging is explained by the increasing number of applications in this spectral range, which includes process monitoring and medical imaging. NIR-to-visible optical upconverters made by integrating a NIR photosensitive unit with a visible emitting unit convert incident NIR light to visible light, allowing imaging of a NIR scene directly with the naked eye. Optical upconverters made entirely from organic and hybrid materials – which include colloidal quantum dots, and metal-halide perovskites – enable low-cost and pixel-free NIR imaging. These devices have emerged as a promising addition to current NIR imagers based on inorganic semiconductor photodiode arrays interconnected with read-out integrated circuitry. Here, we review the recent progress in the field of optical upconverters made from organic and hybrid materials, explain their functionality and characterization, and identify open challenges and opportunities.

## GRAPHICAL ABSTRACT

**Figure:**
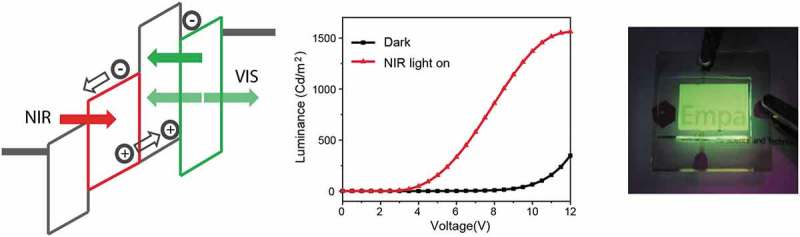

## 1. Introduction

Near-infrared (NIR) light sensing and imaging are of interest due to applications in information and communication technology, passive night vision, optical sensors for tactile contact-free industrial and consumer electronic displays, as well as for robotics and machine vision systems [1–4]. Several commercial applications of NIR imaging are based on the fact that the absorption, reflection and scattering characteristics of molecules can be different for visible and NIR light [5]. Examples include water detection for agricultural food sorting, semiconductor inspection or enhanced vision applications under poor weather conditions, as used in aircraft landing systems or for surveillance and security. Other important applications can be found in the field of bioimaging. When a fluorescent molecule is tagged to the biomolecule of interest, the location of that biomolecule can be monitored externally when illuminated by light. When using fluorescent NIR dyes, the background auto-fluorescence noise of the bio-substrate can be greatly reduced. In addition, reduced optical scattering of NIR light results in a high biological tissue penetration depth that allows for *invivo* imaging applications [6–8].

InGaAs photodiodes are the current standard for NIR imaging, which are typically integrated on complementary metal-oxide-semiconductor readout integrated circuits (ROIC). However, the interconnection is not monolithic and involves complex die-transfer and bonding processes that are not scalable, which limits the pixel size of commercial InGaAs cameras and results in high manufacturing costs [9]. Progress has been made by directly incorporating NIR quantum dot sensitized or organic blend semiconductor photodetectors on top of ROICs, potentially offering an alternative route to sensitive, high-resolution and cost-effective pixelated NIR imaging systems [3,4,9,10].

An approach to pixel-free NIR imaging that avoids any intermediate electronics for data acquisition and processing and that does not require an external display for data visualization is based on so-called NIR-to-visible optical upconversion devices. The basic idea of any upconverter is the serial connection of a NIR photodetector with a visible light-emitting component. When NIR light is absorbed by the photodetector, electron-hole pairs are formed. Under an appropriate bias, photogenerated charges are driven in the light-emitting component leading to visible light emission. The outcome is a NIR-to-visible upconverted image that can be captured using a conventional camera.

The operating mode of upconversion devices is different from the several known photon upconversion processes. Photon upconversion is described as the process that converts two or more sequentially absorbed low-energy photons into a photon of higher energy. Sensitized triplet-triplet annihilation is one such topical example of an efficient optical upconversion process that allows light to be converted into radiation of higher energy at low power excitation. Such materials are developed, i.a., to capture the infrared region of sunlight, thereby increasing the efficiency of photovoltaic cells [11–13].

Early upconverters were made with III-V inorganic compound semiconductors via epitaxial growth or wafer fusion [14,15]. A fundamental issue for monolithic devices involves the need for lattice match between different layers, especially at the photodetector/light-emitting interface. Wafer fusion is a processing technology used to integrate heterogeneous semiconductor materials regardless of their lattice mismatch [16,17]. An advantage of wafer fusion is that defects and interface stress are confined to a few atomic layers at the interface. For pixel-free inorganic upconverters it must be carefully ensured that these devices do not suffer from a low image resolution and contrast because of high lateral charge carrier diffusion [18]. This problem could be partially addressed by replacing the inorganic light-emitting diode with an organic light-emitting diode (OLED) [19]. The integration of an OLED on top of an inorganic photodetector is simple and highly efficient and fast inorganic-organic upconverters have been fabricated. For example, such pixel-free NIR imagers have a spatial resolution of better than 6 μm [20–22]. Inorganic and inorganic-organic upconverters have been reviewed in references [17,23].

Here, we present the status and recent progress in the field of optical upconverters that are entirely made with organic and hybrid materials, including metal-halide perovskites and colloidal quantum dots. A common denominator of these materials is that they can be processed from solution and thin uniform films can be deposited at low cost onto flexible substrates with readily available printing and coating methods. However, it must be mentioned that this process versatility has not been widely exploited for upconverters so far and we are aware of only one reported device that has been fabricated partially from solution, but excluding the top electron-transport, electron-injection and metal electrode layers that were deposited via vacuum evaporation [24].

In addition to manufacturing benefits, organic and hybrid materials have the advantage over broadband absorbing inorganic semiconductors that their optical bandgap and absorption bandwidth can be tailored, to a greater or lesser extent. For the photodetector part in an upconverter, it is particularly desirable to have narrowband NIR-selective sensitive materials that do not absorb in the visible [4,25–28]. Substantial progress is being made with individual organic and hybrid photodetectors, and light-emitting devices [3,4,29–31] and it is anticipated that inspiration can be obtained from these stand-alone devices for the design and realization of improved upconverters. Here, we discuss recent advances and ongoing progress with organic and hybrid optical upconverters both from a materials and device perspective. We outline the operating mechanism and characterization methods of upconverters, summarize the work on state-of-the-art upconverters and identify remaining scientific challenges, such as the realization of solution-processed upconverters, the development of narrowband materials for selective NIR imaging beyond the silicon band edge or concepts for increasing the upconversion efficiency.

## 2. Operating mode and characterization of upconverters

**Figure 1.**
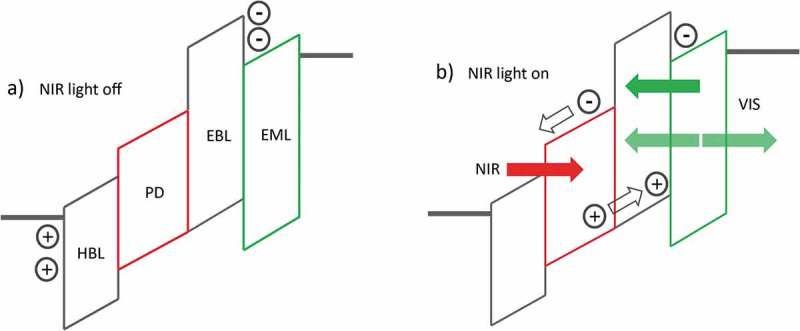
Schematic of a generic optical upconverter in the absence and presence of NIR light. HBL denotes hole blocking layer, PD photodetector, EBL electron blocking layer, and EML visible emitting layer.

Figure 1 shows a schematic cross-sectional view of the most commonly used upconverter architecture to date. The device is a monolithic stack of four layers that are sandwiched between two electrodes: a NIR photodetector, an emitter layer, a hole blocking layer and an electron blocking layer. Not included in the upconverter stack is a frequently inserted hole transport layer between the photodetector and the electron blocking layer. Both in the absence (off state) and presence (on state) of NIR light a voltage bias is applied to the device. In the dark, electrons are blocked at the interface between the electron blocking and the emission layer, and holes are blocked at the interface between the electrode and the hole blocking layer, no current is flowing and therefore no light is emitted. Hole and electron blocking layers help to suppress the dark current but are not mandatory for the fundamental device operation. In the on state, NIR light enters the device through the transparent (substrate) electrode, is absorbed by the photodetector and converted with an external quantum efficiency (EQE_det_) into free charge carriers. Subsequently, electrons are transported via the hole blocking layer to the anode and holes are driven into the emitting layer where they recombine with electrons under the emission of visible light with external quantum efficiency EQE_em_. When a thick metallic mirror-like top electrode is used, the emitted light is out-coupled through the transparent substrate electrode only. In the case of a (semi-) transparent top electrode, light is emitted through both electrodes and can also be detected at the opposite side of the NIR light source.

**Figure 2.**
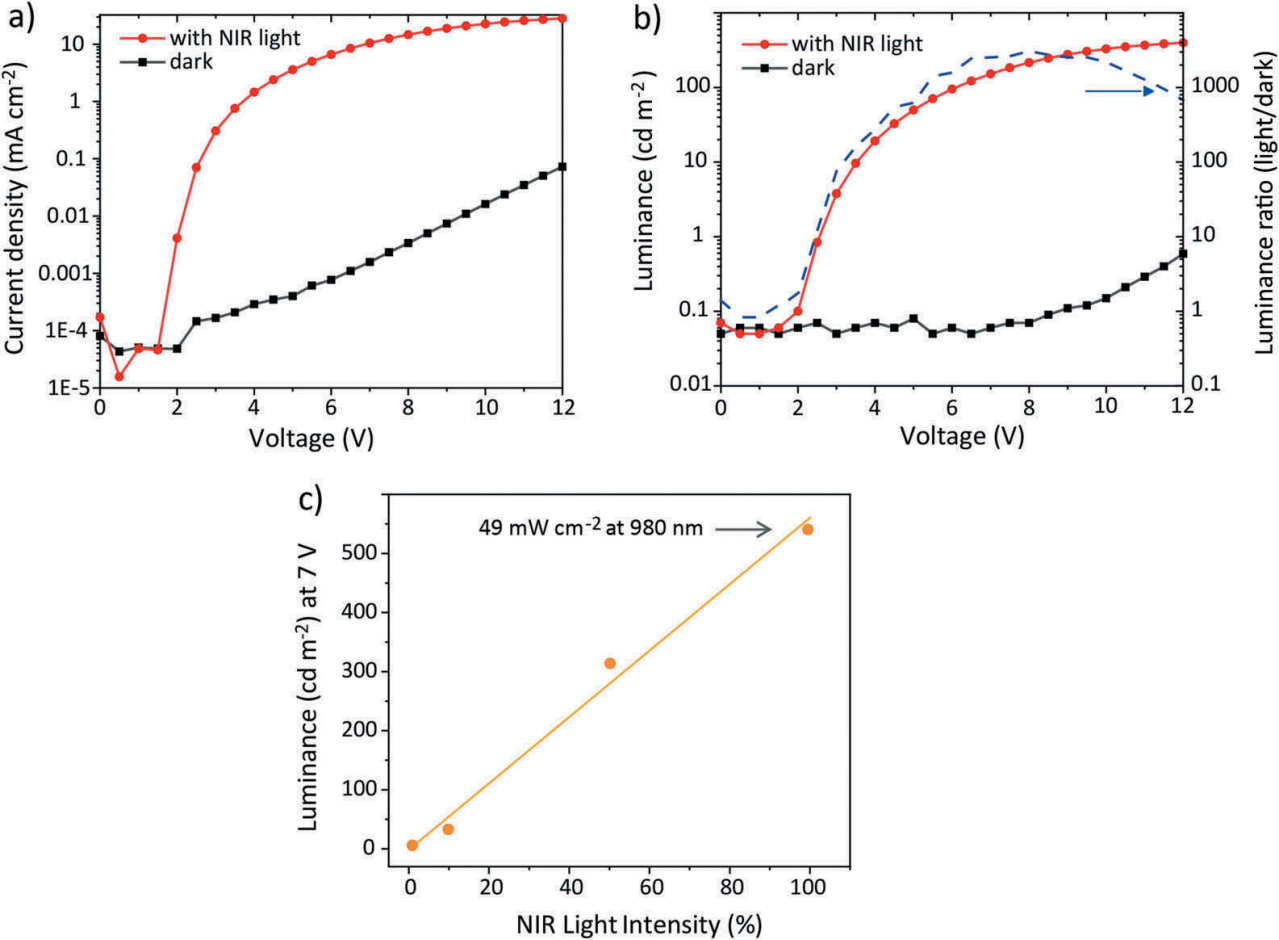
Typical current-voltage (a) and luminance-voltage (b) characteristics with and without NIR light. Data were measured with an upconverter as reported in reference [25]. (c) Luminance of an organic upconversion device (unpublished results) at 7 V for different NIR light intensities. The dark current-induced luminance was subtracted from the total luminance measured in the presence of NIR light.

The upconverter performance can be evaluated by applying a voltage (V) and measuring the current (J) and visible emitted light (L). Typical J-V-L characteristics are shown in Figure 2. A pronounced dark current increase occurs only above ~8 V (Figure 2(a)) resulting in a dark current induced luminance beyond this voltage (Figure 2(b)). In the on state, the current rises sharply at ~2 V and levels off beyond ~6 V. A similar trend is observed for the luminance.

A figure of merit is the luminance on–off ratio (Figure 2(b)), which can also be evaluated for the current. The on–off ratio is small for a small voltage bias where the dark and NIR-light induced luminance levels are small, as well as for a high voltage bias where the dark luminance rises strongly. In between, the on–off ratio passes a maximum at a certain voltage (~3000 at 7 V in Figure 2(b)). It is plausible that a high-performing device should have a low dark luminance and, to minimize the power consumption, the maximum on–off ratio should be at a low voltage. Note that the actual value of the luminance on–off ratio depends on the used NIR light intensity. To achieve the highest image contrast the upconverter can be run at the voltage of the on-off maximum if the luminance level is acceptable for that specific application.

During NIR illumination, the current is limited by the number of charge carriers that are generated in the photodetector and that are injected into the emission layer, which explains the current and luminance saturation. If the NIR intensity is increased, more charges are photogenerated and consequently the luminance level increases. Figure 2(c) shows the luminance when varying the NIR light intensity over two orders of magnitude and the luminance beyond the saturation voltage linearly depends on the NIR light intensity. A linear device response is beneficial for a direct imaging device.

By measuring the number of incident NIR photons and evaluating the number of visible emitted photons, the photon-to-photon conversion efficiency *ɳ_ph_* can be calculated:

(1) $$\eta_{ph}=\frac{N_{out}}{N_{in}}=\frac{\int \frac{I_{out}\left(\lambda \right)\lambda }{hc}d\lambda }{\frac{P_{in}\lambda_{in}}{hc}}=\frac{\int I_{out}\left(\lambda \right)\lambda d\lambda }{P_{in}\lambda_{in}}$$

Here, *N_out_* is the number of visible photons per second emitted into half space, *N_in_* is the number of NIR photons per second impinging on the active device area, *I_out_* is the intensity of visible light emitted at wavelength lambda into half space, *P_in_* is the laser power impinging onto the active device area, and *λ_in_* is the incident NIR radiation wavelength. If a monochromatic laser is used as the NIR light source, *N_in_* can be immediately determined from the measured power of the laser. For the determination of *N_out_*, protocols for the efficiency measurements of OLEDs can be followed [32]. Often, a luminance meter is used to measure the visible emitted light. The dark luminance is superimposed on the NIR-induced luminance and must be subtracted before determining *N_out_*. The meter calculates a value of the surface luminance in forward direction of the total emission spectrum, in cd m^−2^. With the, often not correct [33], assumption that the emission pattern is Lambertian, *viz*. by multiplying by *π*, the emission into half space is then obtained. For *N_out_* the electroluminescence spectrum of the emitting material must also be known. When using a non-transparent top electrode the luminance must be measured from the same side as the incident NIR light. In this case, a fraction of emitted light can be re-absorbed in the photodetector unit thereby changing the (often) known electroluminescence spectrum of a stand-alone light-emitting device [25]. Therefore, it is advisable to measure the electroluminescence spectrum of the upconverter under operation.

An estimate of the photon conversion efficiency can also be obtained from the efficiencies of the individually fabricated photodetector and emitting device via *ɳ_ph_* ≈ EQE_det_ × EQE_em_, but in many cases the true *ɳ_ph_* of the upconverter will be lower because of, for example, hindered charge transport from the photodetection to the emitter unit or re-absorption of visible emitted light.

As shown in Figure 2, both the current and luminance level saturate with increasing voltage and therefore *ɳ_ph_* levels off. In several reported upconverters, however, no clear saturation plateau for the current and luminance was observed and therefore *ɳ_ph_* steadily increased with increasing voltage. In such a case, a higher value of *ɳ_ph_* can be obtained when increasing the voltage but this then has the disadvantage that also the dark luminance will increase strongly; a high voltage might also be incompatible with the conceivable requirement that a simple low-voltage battery should be able to drive a low-cost or portable upconversion device.

Organic and hybrid upconverters are particularly suitable for single-element imaging, meaning that no pixel structure is needed to isolate photogenerated current. The output and input images are spatially correlated, and lateral current spreading must be suppressed to maintain the spatial correlation. Due to the poor lateral conductivity and very thin thickness of the active layers, lateral current spreading in organic and hybrid upconverters is small, but no rigorous analysis of the achievable spatial resolution of such NIR imaging devices has been reported.

Finally, an important figure of merit of an upconverter is the time delay between the NIR input and visible output light. The response speed determines the frame rate for image generation and depends on parameters such as the electroluminescence lifetime of the emission layer, the resistance-capacitance time constants of the devices (which will be lower for smaller devices) and the charge-carrier mobility.

## 3. All-organic upconverters

**Figure 3.**
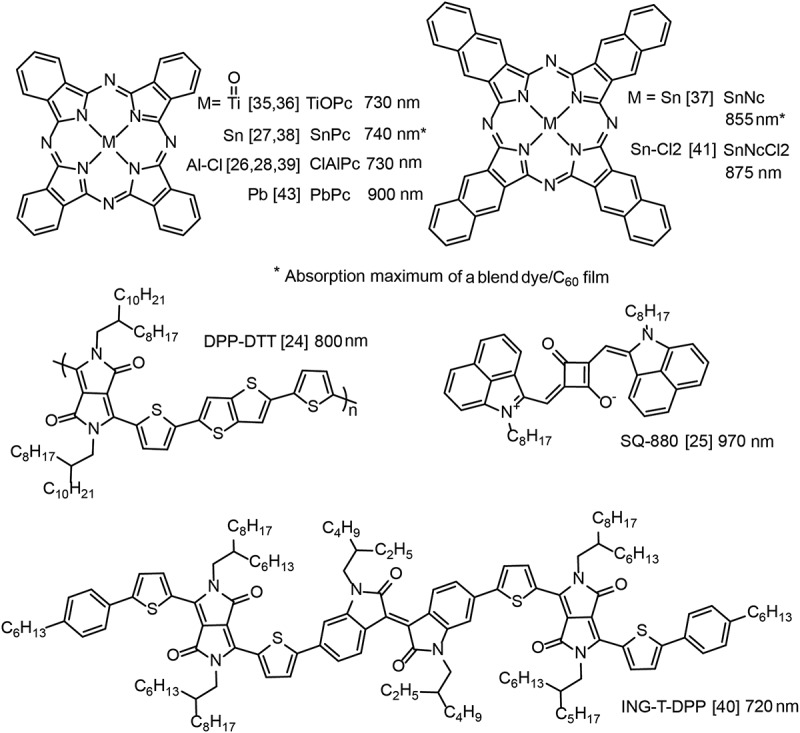
Selected examples of organic NIR sensitizers used in optical upconverters. The indicated wavelengths denote film absorption maxima, as stated in the references or read out from the displayed absorbance spectra. Abbreviated designations for organic compounds were taken from the references.

Several all-organic upconverters (sometimes abbreviated as OUCs or OUDs) used a NIR organic photodiode in combination with an OLED based on fluorescent materials [25,34–37]. An organic upconverter consisting of a single titanyl phthalocyanine (TiOPc, see Figure 3) absorber layer on indium tin oxide (ITO) was combined with an OLED containing tris-(8-hydroxy quinoline) aluminium (Alq_3_) as the emissive layer including a hole transporting layer, and light at 650 nm was upconverted to green light at 530 nm [35]. A TiOPc film absorbs in a broad range of 600–900 nm. This was exploited in subsequent work, and the same TiOPc photosensitizer was combined with a blue OLED to upconvert light at 780 nm to 470 nm [36]. In the dark, the luminance turn-on was at 12 V, while in the presence of NIR light turn-on was at 5 V and the maximum on–off ratio was about 1000 at 12 V. The electroluminescence response time to an NIR light trigger was about 300 μs.

A blend layer of tin naphthalocyanine (SnNc):C_60_ was used as NIR sensitizer in an organic upconverter together with an Alq_3_ OLED [37]. The peak absorption of SnNc is at 875 nm, red-shifted by ~130 nm compared to phthalocyanines. NIR light at 830 nm was successfully upconverted, but the device showed a rather large dark luminance level because no hole blocking layer was used.

A photodetector with peak sensitivity at 980 nm and an internal photon-to-current conversion efficiency of close to 100% under reverse bias was demonstrated with a NIR-selective squaraine dye (SQ-880):PC_61_BM blend, where PC_61_BM denotes [6,6]-phenyl-C_61_-butyric acid methyl ester [25]. The photodetector was integrated in an upconverter by using a MoO_3_ hole transport layer to facilitate charge injection into the Alq_3_ emitter. The materials of this organic upconverter absorb very little in the visible. By using a semitransparent top electrode, visibly transparent devices could be fabricated with an average visible transmittance of 65% and a peak transmittance of 80% at 620 nm.

**Figure 4.**
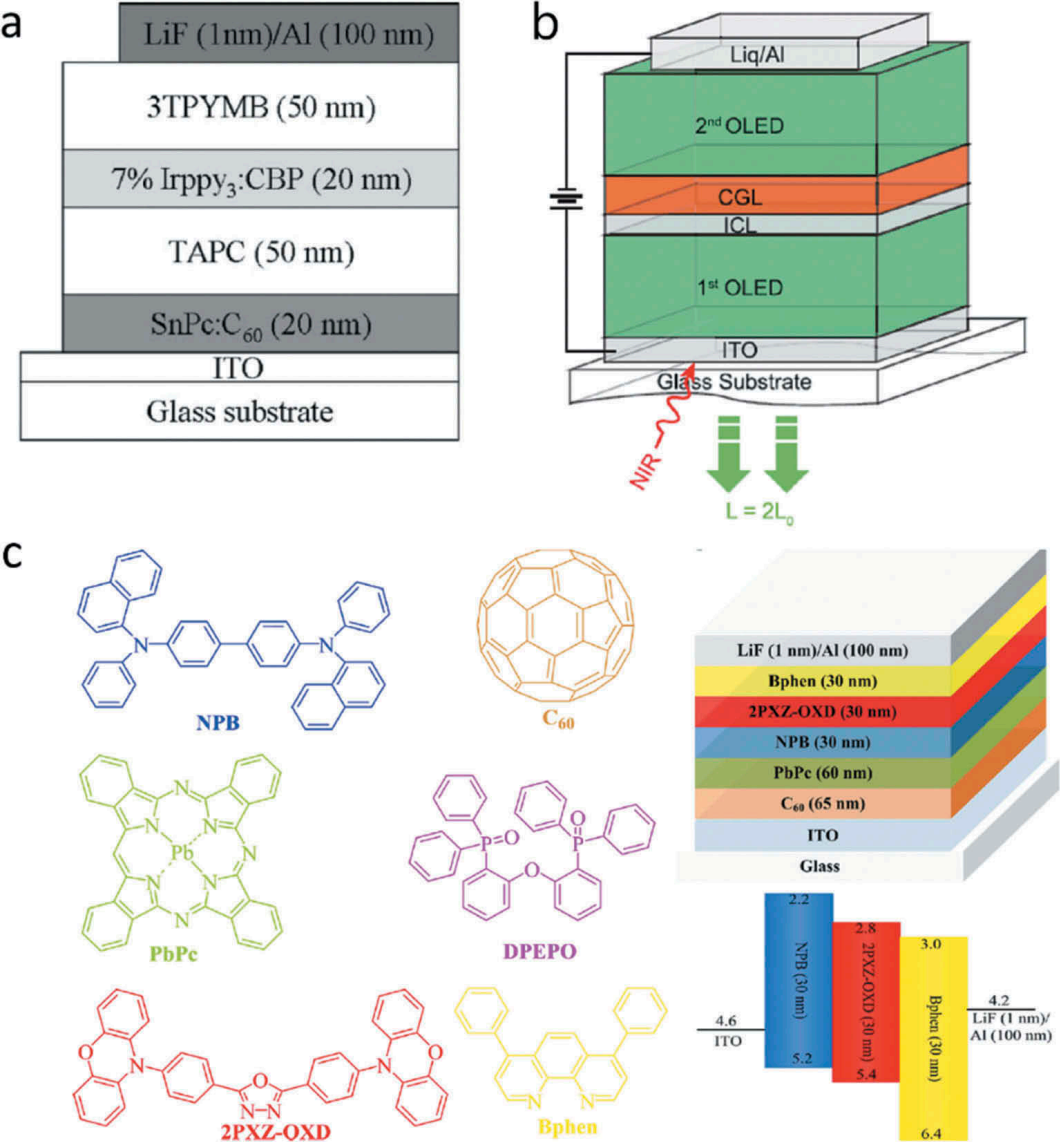
Selected examples of upconverter layer stacks taken from the literature. a) The first example of an all-organic upconverter with a NIR photon-to-visible photon conversion efficiency higher than 1%. The chemical structure of the NIR-absorbing tin phthalocyanine (SnPc) is shown in Figure 3. TAPC denotes 1,1-bis[(di-4-tolylamino)phenyl]cyclohexane, Irppy_3_:CBP denotes fac-tris(2-phenylpyridinato)iridium(III)-doped 4,4-N,N-dicarbazole-biphenyl and was the phosphorescent emitter layer, 3TPYMB denotes tris[3-(3-pyridyl)-mesityl]borane [38]. b) Scheme of a tandem upconverter with two OLED units. CGL denotes charge-generation layer, ICL is a thin intermediate connecting layer that assists the photoelectron injection into the first OLED. Reproduced with permission from Ref [41], Copyright 2018 AIP; c) Molecular structures, device layer stack and schematic energy level diagram of an upconverter with a photon conversion efficiency of over 100%. Reproduced with permission from Ref. [43], Copyright 2018 ACS.

A marked efficiency improvement was achieved by using a NIR photosensitizing tin phthalocyanine (SnPc):C_60_ bulk heterojunction photodiode and an OLED based on a phosphorescent material (see Figure 4) [38]. When irradiated with NIR light at 830 nm, the device turned on at 2.7 V and the luminance continuously increased with increasing operation voltage. The highest luminance on/off ratio was at 12.7 V. *ɳ_ph_* was ~1% at 10 V, while *ɳ_ph_* (max) was 2.7% at the maximum voltage of 15 V. This value is in the expected range, when estimated from the individual photodetector and OLED efficiencies, *ɳ_ph_* ≈ EQE_det_ × EQE_em_ = 15% × 20%.

The intrinsic *ɳ_ph_* potential of that upconverter was not fully harnessed at that time because the absorption maximum of the sensitizing SnPc:C_60_ layer is at 740 nm and a relatively small fraction of incident light at 830 nm was absorbed in the tail of the long wavelength absorption band [38]. Indeed, it was demonstrated later on that the peak photon conversion efficiency is at 740 nm and *ɳ_ph_* = 11.3% was measured at 12 V [27]. The device structure was at the same time improved by adding a hole blocking layer between ITO and the NIR sensitizer.

An organic upconverter composed of a chloroaluminium phthalocyanine (ClAlPc):C_70_ photodetector and a CBP/Irppy_3_ OLED was demonstrated under NIR illumination at 780 nm [26]. The optimum ClAlPc:C_70_ blend ratio was 1:5 for the stand-alone photovoltaic device, but in the upconverter the best ratio was at 3:1. A reduction of the C_70_ content in the photodetector part was also beneficial because C_70_ absorbs in the visible and therefore re-absorbs light emitted by the OLED. This upconverter showed a very efficient hole injection from the photodetector into the OLED and a steep current and luminance rise after turn-on. The luminance at 2.7 V was already at 100 cd m^−2^ and the maximum of the current on–off ratio was at 3 V. *ɳ_ph_* was ~4% at 7 V. The device was used to take 3D images of real objects in a night-vision demonstration. Subsequently, with a similar ClAlPc:C_60_ sensitizer and the CBP/Irppy_3_ emitter, the dark current of the upconverter could be further reduced and the on–off ratio increased by tuning the Irppy_3_ doping content [39].

In addition to fluorescent and phosphorescent OLEDs, thermally activated delayed fluorescence (TADF) emitters were also tested for organic upconverters [40]. By using different TADF materials, the colour of the upconverted light could be tuned from red to blue. The photon conversion efficiency of these upconverters was rather modest (*ɳ_ph_* below 0.1%) due to the low responsivity of the photodetector, which was composed of a blend of an isoindigo-diketopyrrolopyrrole-based low bandgap material (ING-T-DPP) and PC_61_BM.

In the upconverters discussed so far, holes from the photodetection unit are injected in the OLED where they recombine under light emission with electrons from the cathode (see Figure 1). Also, the reversed architecture is conceivable where photogenerated electrons are injected into the OLED. This device concept was demonstrated in reference [28] where a CBP/Irppy_3_ OLED on ITO was connected with an overlying ClAlPc:C_60_ photodetector. In such a layer stack, however, a considerable energetic barrier for electron transfer from the photodetector to the electron transporting layer of the OLED exists. Key to the device functioning was to insert a thin LiF(1 nm)/Al(1.5 nm) electron injection layer between the photodetector and the OLED. Thereby, NIR light at 780 nm could be upconverted with *ɳ_ph_* = 3.46% and the device turned on at a low voltage of 2.27 V. A system composed of the organic upconverter and a commercial digital camera was used to produce visible images of blood vessels.

**Figure 5.**
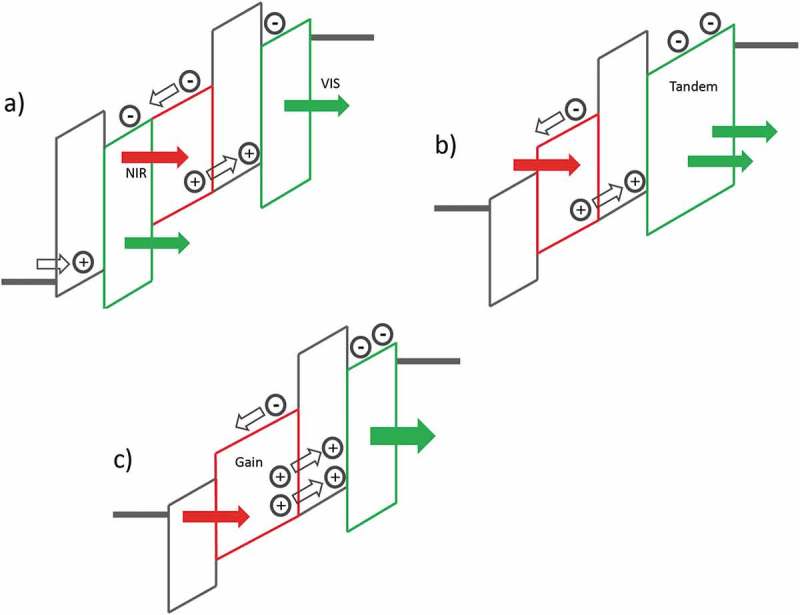
Layouts of upconverter architectures with increased NIR-to-visible photon conversion efficiency. Several crucial charge-blocking, -transporting and -generating interfacial layers are omitted in these simplified pictures for clarity. a) In a tandem upconverter the photodetector is sandwiched between two emitter layers, electrons, as well as holes, are harvested to generate visible light. b) A tandem OLED emits two visible photons per one photogenerated charge. c) A photodetector with a gain injects more than one hole into the emitter layer per one photogenerated charge.

Taking a step forward, a tandem organic upconverter was designed by sandwiching a charge generation layer between two phosphorescent OLEDs (see Figure 4) [41]. The idea of a tandem upconverter is that the photogenerated electron is driven in the first OLED, and the photogenerated hole is driven into the second OLED. Compared to a conventional upconverter where only the holes are used for current-to-light conversion, the efficiency of a tandem upconverter can thereby in principle be increased by a factor of two (see Figure 5). A thin intermediate layer of Liq(1 nm)/Al(1 nm) was added to facilitate electron injection into the first OLED, a similar approach as used in reference [28]. The NIR sensitizer was tin(IV)2,3-naphthalocyanine dichloride (SnNcCl_2_), either used as a single layer, an SnNcCl_2_:C_60_ bulk heterojunction or as a C_60_:SnNcCl_2_ bilayer. Compared with the control device the efficiency of the tandem devices was indeed better by a factor of around 3.5 and *ɳ_ph_* = 5% was achieved, however only at a high voltage of around 25 V. The turn-on voltage (~7 V) under NIR illumination was also rather high. These results suggest that despite the use of a charge injection layer a large energy barrier for electron injection into the first OLED remained.

Other device concepts than the tandem upconverter have been proposed to overcome the efficiency limitations of a conventional upconversion device. In one approach, a broadband photodetector was combined with a tandem phosphorescent OLED [42]. A tandem OLED emits two photons per injected charge which increases *ɳ_ph_* by a factor of two when integrated in an upconverter (see Figure 5). Note, however, that the power efficiency (lm W^−1^) of a tandem OLED is not increased compared to a single-unit OLED, meaning that the operating voltage is higher. An additional efficiency increase, to a maximum of *ɳ_ph_* = 29.6%, was attributed to a photomultiplication effect by visible emitted light that was re-absorbed by the photodetector, resulting in additional electron-hole pairs. However, a noteworthy disadvantage is that broadband photodetection result in a nonselective NIR response with the consequence that the upconversion device is in the on state even in the sole presence of visible incident light.

An organic upconverter with *ɳ_ph_* over 100% was demonstrated by exploiting a photomultiplying effect of a NIR photodetector (see Figure 4) [43]. For an ITO/lead phthalocyanine (PbPc)/C_60_/Al photodetector under 10 V reverse bias and for a light intensity of 0.052 mW cm^−2^ at 808 nm, EQE_det_ was 1.95 × 10^4^%, demonstrating a current gain mechanism due to trap-assisted photomultiplication (see Figure 5). EQE_det_ decreased strongly for higher NIR light intensities. This photodetector was then integrated with a TADF OLED into an organic upconversion device. The photodetector gain translated into a high upconversion performance and *ƞ_ph_* was ~150% at 15 V. The response speed of the upconverter at 3 V was measured to be 118 μs.

## 4. Hybrid upconverters

There are few upconverter examples that have made use of hybrid materials, either in the photodetector or in the emitter unit. In a first example, a colloidal PbSe nanocrystalline film was used as an NIR sensitizing layer [44]. The PbSe photodetector was integrated with a CBP/Irppy_3_ OLED via a hole transporting layer composed of 1,1-bis[(di-4-tolylamino)phenyl]cyclohexane (TAPC). Addition of a ZnO hole blocking layer suppressed the dark current density by two orders of magnitude. In the presence of NIR light, device turn-on was between 7 and 8 V, a relatively high voltage compared to all-organic upconverters discussed above. The PbSe upconverter showed sensitivity up to 1500 nm and a maximum at 1300 nm with *ɳ_ph_* = 1.3%, at an operating voltage of 17 V. The low value of *ɳ_ph_* was attributed to the small fraction of NIR light absorbed in the 50 nm thick PbSe film. Subsequently, a similar upconverter was fabricated by using PbS quantum dots as NIR sensitizing film with a peak sensitivity at 1200 nm [27]. This device was incorporated in a digital camera and an imaging system capable of NIR as well as visible imaging was realized.

**Figure 6.**
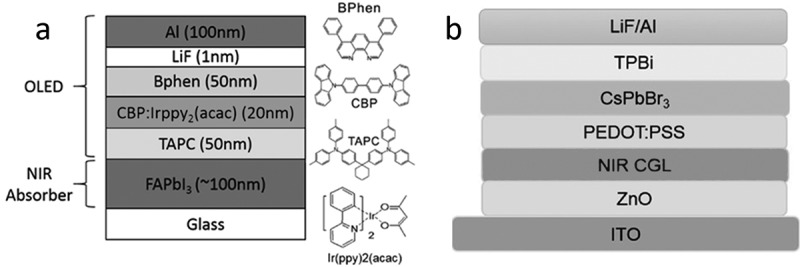
Examples of hybrid upconverter layer stacks taken from the literature. a) Device architecture of an upconverter using formamidinium lead iodide as a NIR absorber integrated with a phosphorescent OLED [45]. b) Layer stack of an upconverter consisting of an organic NIR charge generation layer (CGL) and a perovskite LED. TPBi denotes 2,2ʹ,2ʹ’-(1,3,5-benzinetriyl)-tris(1-phenyl-1H-benzimidazole). The four layers from ZnO to CsPbBr_3_ were processed from solution [24].

Formamidinium lead iodide (FAPbI_3_) as a sensitizer for NIR wavelengths below ~830 nm was integrated with a phosphorescent OLED (see Figure 6) [45]. The sensitizer layer thickness was limited to below 200 nm due to the strong absorption of the perovskite in the visible, resulting in considerable re-absorption of upconverted light. The upconverter was characterized by an efficient hole injection from the perovskite layer via the hole transporting layer TAPC to the OLED and a steep luminance rise after turn-on at 1.9 V. This voltage was below the turn-on voltage of the reference OLED, which was attributed to the photovoltaic effect of the perovskite layer, supplying an additional voltage to the OLED. *ɳ_ph_* for NIR light at 800 nm was 3% at a low voltage of 5 V. The luminance response was linear over a NIR light intensity range corresponding to at least 24 dB. The rise time and the fall time of the upconverter in response to NIR light pulses at 810 nm were 10 and 43 μs, respectively.

Alternatively, a CsPbBr_3_ perovskite LED with a narrow emission at 520 nm was used for an upconverter (see Figure 6) [24]. Interestingly, this device was processed mostly from solution except for the upper electron transport layer/LiF/Al top contact, which was deposited by thermal evaporation. The solution-processed layer stack was ZnO/NIR-photodetector/PEDOT:PSS/CsPbBr_3_, where the NIR-photodetector was an organic bulk heterojunction layer composed of a low bandgap polymer (DPP-DTT) and either PC_70_BM or a non-fullerene acceptor, and PEDOT:PSS (poly(3,4-ethylenedioxythiophene)-poly(styrene sulfonate)) was used as hole transporting layer. Upconverters with PC_70_BM showed a large dark current and consequently a low luminance on–off ratio. In addition, PC_70_BM absorbs a fraction of emitted light which decreased the luminance further. The non-fullerene acceptor used absorbs little in the visible and the device performance metrics, such as a luminance on–off ratio of ~400 (5 mW cm^−2^ at 850 nm) and *ɳ_ph_* = 1.9% at 6 V were improved. The transient current response to NIR light of this upconverter was 76 μs.

## 5. Challenges and outlook

A great deal of progress has been made with organic and hybrid optical upconverters over the last years, but it is clear that a number of challenges must be resolved to advance the field and to finally transform present-day prototype NIR imagers into a technology. Commercial InGaAs cameras are very expensive, which prevents their widespread use in most consumer and low-end applications. True cost benefits and a wider application range can be achieved when upconverters are fabricated entirely from solution using high-throughput printing and coating processes. To date, most upconverters were fabricated entirely via thermal evaporation processes or by a combination of solution processing and thermal evaporation, which from the point of view of the required equipment and production processes on two levels simultaneously should probably be avoided.

Key to multilayer solution processing is to have a layer structure that can withstand solvents used in subsequent processing. This difficulty is well known from the field of solution-processed single OLEDs and photodetectors, and concepts such as the use of orthogonal solvents and cross-linkable layers can be adopted [46–49]. Therefore, there seems to be no fundamental barrier to the realization of solution-processed upconverters in the near future. A critical challenge for solution-processed optoelectronic devices, in general, is the deposition of the charge-injecting/top electrode layer. For OLEDs and photodetectors fabricated in the inverted architecture, a solution-processed top PEDOT:PSS layer works fine and is optionally supported by a conductive metal [50–52]. However, upconverters presented so far were built in a regular architecture and electron injection takes place at the top electrode. A number of solution-processable electron injection materials as a replacement for the typically evaporated alkali metals have been described [53]. A promising approach was the use of a ZnO/polyethylenimine ethoxylated (PEIE) between a top Al cathode (evaporated) and the emitting organic layer in OLEDs [54]. A final processing challenge is that in an upconverter the top electrode should preferably be visibly transparent, in order that the emitted visible light can be detected at the opposite side of the incident NIR light. So far, semitransparent cathodes of upconverters were deposited as thin metal/dielectric layer via thermal evaporation [25–27]. However, several concepts for solution-processable transparent electrodes can be borrowed from related fields of research, such as spray-coating of metal nanowires or lamination of carbon nanotubes and graphene [55,56].

From a materials perspective, it is clear that the vast opportunities that hybrid materials can offer for advanced upconverters have not been exploited so far. All-hybrid upconverters or devices with narrow band emitting colloidal quantum dot LEDs are interesting but so far unexploited device concepts. For photodetectors there exists great demand for selective NIR absorbing materials with a narrow spectral response beyond the silicon band edge, which is around 1100 nm. Upconverters with sensitivity above that wavelength present a unique selling point because light between ~650 nm and 1100 nm, although not visible to the human eye, can also be detected with many digital cameras. The red, blue and green colour filters on the photosensors of the camera are to some extent sensitive in the NIR range and a filter that blocks NIR light from reaching the sensor is added. However, if this filter does not block the NIR light completely, even an unmodified standard digital camera is capable of capturing NIR light. The NIR sensitivity can be easily tested by pointing a TV remote control on the camera and pushing one of the buttons. No visible light can be seen, but on the camera’s screen, the NIR light emitted by the device appears as a bright spot. For deliberate NIR imaging with digital cameras, the NIR blocking filter can be removed and replaced by a filter that blocks the visible light. Therefore, a commercial digital camera can be used as a NIR broadband image sensor up to 1100 nm, well suited for many application cases.

Colloidal quantum dots can have a response above 1100 nm but in many cases have a broad absorption spectrum in the visible [57,58]. Also, few organic photodetectors with a spectral response above 1100 nm have been reported but again mostly using materials that absorb also in the visible [59–61]. There are several reasons why the photodetector should not respond to visible light. First, selective NIR absorbers are needed because visible light absorption of broadband photodetectors results in a non-selective NIR response. In addition, NIR selective absorption is important for upconverters that contain a non-transparent top electrode. In such a case, the incident NIR light and out-coupled visible light is measured from the same side and a broadband photodetector can re-absorb a fraction of the visible light emitted. Furthermore, the majority of organic photodetectors still use a donor/fullerene acceptor materials combination. This is problematic because fullerenes possess significant blue-green absorption. Intrinsic photoinduced charge generation in single organic layers or the use of non-fullerene acceptors are possible approaches to solve this problem [24,62,63]. Finally, when both the photodetector and emitter materials do not absorb in the visible and when using transparent electrodes, visibly transparent devices can be fabricated that are interesting for glass-integrated electronics or imagers that can simultaneously detect incident and NIR upconverted visible light [25–28].

In addition to visible transparency, applications such as machine vision systems or spectral biological imaging require NIR sensitizer with a narrow spectral response [1,4]. One approach for organic narrowband photodetectors is to use very thick organic films in which the response is narrowed (<20 nm) to the absorption onset, leading to visible blind and tuneable red and NIR narrow band response [2,64]. The idea of this charge-collection narrowing approach is that the above-bandgap wavelength photons are absorbed close to the transparent electrode and these generated charges recombine and cannot be collected, whereas the long wavelengths generated charges are more homogeneously distributed through the layer and these charges can be collected. When integrated in an upconverter, such charge-collection narrowing photodetectors will completely absorb the visible emitted light and the upconverted light must be measured at the opposite side of the incident NIR light; this requires a semitransparent top electrode. Alternatively, narrowband NIR response up to 1550 nm has been reported based on enhanced intermolecular charge-transfer absorption using an optical microcavity [1]. The concept was shown with materials that absorb in the visible but when using transparent donor/acceptor combinations, visibly transparent photodetectors can potentially be obtained.

Figure 3 shows selected examples of organic NIR sensitizers integrated in upconverters to date. All sensitizers have a single maximum absorption in the NIR but cannot be designated as true NIR narrowband absorbers because the absorption bandwidths are well over 100 nm and slightly extend into the visible. It is obvious that the catalogue of tested organic NIR sensitizers for upconverters is very limited. Other candidate dyes with absorptions above ~750 nm are croconic dyes, organic metal complexes, donor-acceptor charge transfer chromophores or extended π-conjugated chromophores, but it should be mentioned that some of these dyes are difficult to synthesize, have poor solubility, or low molar absorptivity [65–70]. An interesting class of NIR dyes for further investigation is cyanines. Cyanine dyes are synthetically relatively easy accessible, have very high light extinction coefficients and good film-forming properties [71]. Thin films of heptamethine cyanines appear almost colour free in the visible and have been used, e.g., for the fabrication of transparent solar cells and photodetectors [72–74]. Recently, cyanine dyes with photosensitivity up to 1600 nm have been reported [75]. Furthermore, it is well known that cyanine dyes can self-assemble into highly ordered (J or H)-aggregated nanostructures. The formation of J-aggregate results in a redshift and narrowing of the absorption band which has been exploited for a NIR narrowband cyanine photodetector [76]. These results suggest that cyanines are an interesting dye class capable of realizing true narrowband upconverters with photoresponse beyond the silicon band edge.

From the point of view of performance, there is certainly a demand for increased *ɳ_ph_*. In most cases, NIR light with an intensity of several (tens of) mW cm^−2^ was upconverted but very efficient devices have already been demonstrated. For example, NIR light at 808 nm with an intensity of 52 μW cm^−2^ was upconverted to green light with a maximum luminance on–off ratio of about 60 [43]. From the linear dynamic NIR photoresponse range, it was estimated in reference [42] that organic upconverters are capable of detecting NIR light intensities down to 10 μW cm^−2^. It was also demonstrated that these upconverters have a lower light detection limit than commercial NIR detection cards made from rare metals.

Further development of upconverters that include a photodetector with a gain is a promising approach to increasing the device efficiency [43] because this approach keeps the most simple layer stack architecture. In a photodiode, EQE is typically limited to a maximum of 100%. By using trap-assisted photomultiplication, photodetectors with much higher EQE values were demonstrated. In one example, ZnO nanoparticles were blended with semiconducting polymers [77]. ZnO acts as an electron trap under illumination; this redistributes the energy levels and results in a strong secondary hole current through the polymer. The device changed from a photodiode in the dark to a photoconductor under illumination, and impressive values for the EQE (>3×10^5^%) and detectivity (3.4×10^15^ Jones) were measured. Similarly, trap-assisted photomultiplication was obtained in PC_71_BM-doped (1%) P3HT (poly(3-hexylthiophene-2,5-diyl)):PC_71_BM photodetectors (EQE 16,700%) [78]. The concepts of charge-collection narrowing and trap-assisted photomultiplication were combined to fabricate narrowband photodetectors with a gain [79,80]. A challenge of this approach is that long-lived trap states reduce the frequency response, compromising the frame rate of upconverters with a gain.

Finally, a photocurrent gain in the photosensitive unit of an upconverter could also be realized with phototransistors. In one example, a quite tricky assembled PbS quantum dot based vertical phototransistor with a gain was combined with an OLED [81]. The device displayed a high *ɳ_ph_* of over 1000% at low (<3 μW cm^−2^) infrared power densities, and *ɳ_ph_* was about 20% for a light intensity of ~75 μW cm^−2^. In an alternative upconverter device architecture with photon-to-electron gain, a NIR absorption layer was incorporated between the carrier transport layer and the emission layer in heterostructured organic light-emitting field effect transistors [82]. *ɳ_ph_* was 28.7% for a NIR light intensity of 10.4 μW cm^−2^ and 0.93% for 196 mW cm^−2^. Due to the device layout, this device is probably less suited for large-area direct imaging applications.

**Figure 7.**
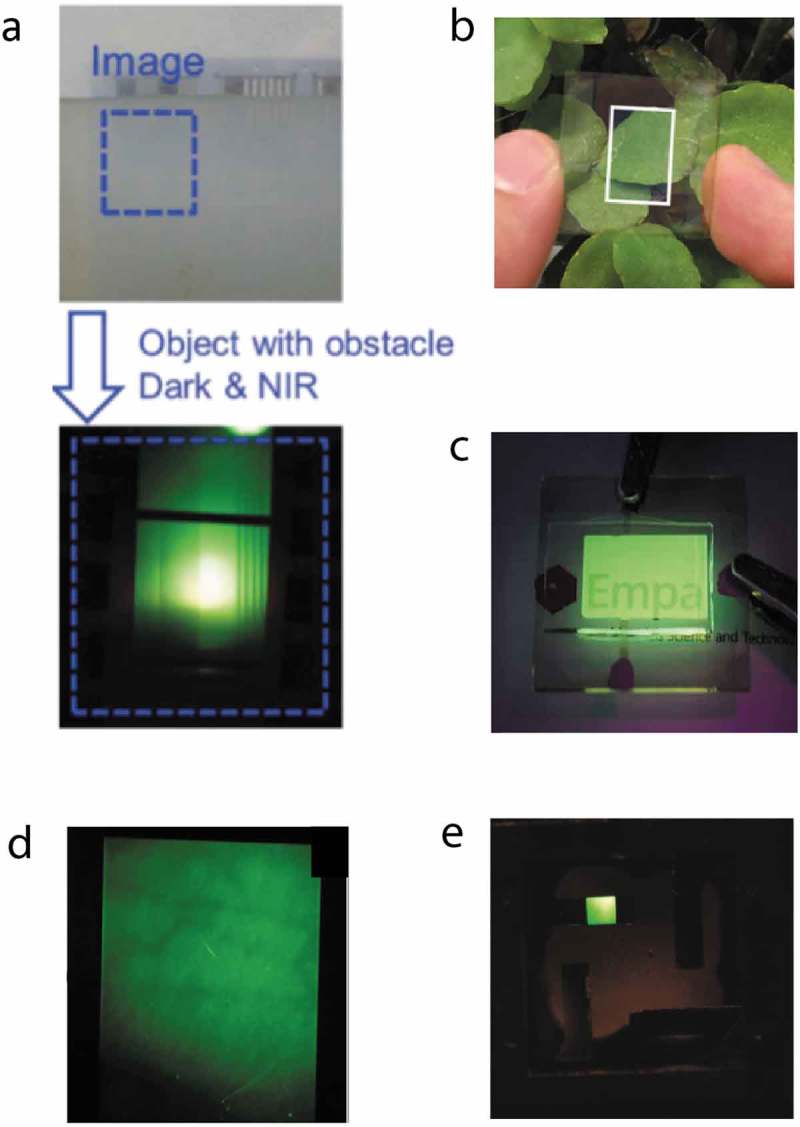
Selected upconversion examples taken from the literature. a) Visible and upconverted image of a metal mask positioned behind turbid water, showing that turbid water is transparent to NIR light. Reproduced with permission from Ref. [26], Copyright 2015 Wiley; b) Photo, and c) emitted green light of a see-through all-organic upconversion device, including a transparent top and bottom electrode. Reproduced with permission from Ref. [25], Copyright 2018 ACS; d) Upconverter image of a human forearm with observable subcutaneous blood vessels. Reproduced with permission from Ref. [28], Copyright 2016 Nature; e) Example image made with a colloidal PbSe nanocrystal photodetector combined with a green-emitting OLED, light at 1300 nm was upconverted. Reproduced with permission from Ref. [44], Copyright 2011 ACS.

Figure 7 compiles some upconversion examples and images taken from the literature.

Figure 7(a) demonstrates that imaging in the NIR can be used for vision enhancement in poor weather conditions. The top image was taken in the visible and shows part of a metal mask that was placed behind a vessel filled with turbid water. In the lower image, NIR light was directed through the vessel onto the mask. The reflected NIR light from the mask was upconverted after passing again through the turbid water, and the image was captured with a conventional digital camera. NIR light passed the turbid water with little distortion, and the metal mask could clearly be detected. Figure 7(b) shows a photo of a transparent organic upconverter with a high average visible transmittance of 65%. In Figure 7(c), this upconverter was placed in front of a company logo in a dark room. When irradiated with NIR light at 980 nm, the upconverter emitted green light and because of the device transparency, the logo could clearly be read. Figure 7(d) shows an upconverted image of a human forearm. NIR light was directed onto the forearm and the backreflected NIR light was upconverted. NIR light penetrates the human skin better than visible light. What is clearly visible in this image are darkened lines that indicate the position of subcutaneous blood vessels. Image contrast is due to a combination of scattering in the tissue and absorption by the blood vessels [5]. When illuminating the forearm with visible light, these features were not apparent.

## 6. Conclusions

Optical upconversion devices based on organic and hybrid materials have progressed significantly as a result of advances in materials science and device design. The realization of truly low-cost NIR imagers opens novel and alternative possibilities for optical sensing applications that cannot be covered by the current, inorganic elemental semiconductor technology. From novel narrowband NIR sensitizers to upconverters with long operating lifetimes and system integration, a broad range of scientific opportunities and challenges lies ahead to achieve this goal and to eventually transform present-day prototype organic and hybrid upconverters into a technology.
